# Haematological patients admitted to icu: differences between survivors and non-survivors

**DOI:** 10.1186/2197-425X-3-S1-A250

**Published:** 2015-10-01

**Authors:** J Lujan Varas, L Alcázar Sánchez-Elvira, P Villa Díaz, B Llorente Ruiz, R Molina Montero, M Rascón Risco, C Arenillas Juanas, M Manso Álvarez, Y Ortiz de Zárate Ansótegui, JA Cambronero Galache

**Affiliations:** H Principe de Asturias, Madrid, Spain

## Intr

Patients with haematological malignancies admitted to ICU have high mortality. Reticence of intensive care providers to admit and treat these patients is well described in literature.

## Objectives

To evaluate differences between survivors and non-survivors and provide possible independent risk factors for ICU mortality.

## Methods

Single centre observational retrospective study in a 14-bed Intensive Care Unit of a University Hospital. All haematological patients admitted between January-2009 and December-2014 were enrolled. Data acquired included: demographics characteristics, haematological diagnosis, reason of ICU admission, severity-of-illness scores (APACHE and SOFA) and intensive care therapy (mechanical ventilation (VM), extrarenal therapy depuration (ETD) and vasopressor support (VS)).

## Results

We included 38 patients in the study,15(39,47%) were survivors and 23(60,52%) were non-survivors. Median age of 50,47 ± 13,98 vs 59,78 ± 14,73 (p > 0,05) and predominance of males in both groups (60% vs 73,9%, p > 0,05), respectively. In both groups non-Hodgkin lymphoma was the most frequent haematological malignancy, 53% and 30,4 %, survivors and non-survivors respectively and acute respiratory failure was the most frequent reason for ICU admission(66% and 39,1%, respectively). Intergroup comparisons revealed statistically significant differences in APACHE (19,73 ± 8,05 vs 26,48 ± 8,74, p < 0,05) and SOFA (9 ± 3,4 vs 11,83 ± 3,23, p < 0.05). During the first 24h of ICU admission, 60% of the survivors patients had 2 or more organ failures, and 73,9% in non-survivors group. During evolution in ICU, survivors patients required VM and VS in 80% and 66,7%, respectively. None of them needed EDT. Non-survivors required VM and VS in 91% and 95,7% respectively, and 17,4% needed EDT. There were no statistically significant differences in ICU support therapies between survivors and non-survivors. No independent risk factors for mortality were found by logistic regression analysis.

## Conclusions

Mortality in patients with haematological malignancies remains high. There were significant differences in severity-of-illness scores during the first twenty-four hours of ICU admission between survivors and non-survivors. No significant differences in intensive care therapy were found between groups during ICU hospitalization.
